# Non-invasive wood moisture sensing for arthropod infestation prevention using a circularly polarized high-isolation antenna system

**DOI:** 10.1038/s41598-026-58316-1

**Published:** 2026-06-23

**Authors:** Abdelkarim S. Elhenawy, Ahmed Allam, Haruichi Kanaya, Adel B. Abdel-Rahman

**Affiliations:** 1 Electronics and Communications Engineering Department, University of Science and Technology, Alexandria, 21934 Egypt; 2 Electrical Engineering Department, Faculty of Engineering, Kafrelsheikh University, Kafrelsheikh, 33516 Egypt; 3 Graduate School of Information Science and Electrical Engineering, Kyushu University, Fukuoka, 819-0395 Japan; 4 Electrical Engineering Department, Faculty of Engineering, South Valley University, Qena, 83523 Egypt

**Keywords:** Antenna sensor, Moisture content, Microstrip Antenna, Circular polarization, Arthropods prevention, Wood protection, Ecology, Ecology, Engineering

## Abstract

This paper presents a novel circularly polarized microstrip antenna system for non-invasive detection of moisture in wood, enabling early identification of arthropod infestation areas before structural damage occurs. The proposed system consists of two microstrip antennas with opposite polarization directions (LHCP/RHCP) for transmission and reception. The circular polarization direction difference between the transmitter and the receiver allows long distance measurement and ensures low coupling between the antennas; thus, high isolation is maintained when no moisture is present. Moisture presence alters this condition, increasing the received power by 12.5 dB. The antenna system is fabricated on a low-cost FR4 substrate with compact dimensions of 50 mm × 50 mm × 1.6 mm and operates at 2 GHz. Comprehensive simulations and experiments demonstrate reliable operation across multiple conditions, including moisture levels (0–100%), temperature (− 20 °C to 90 °C), antenna positioning, wood type (Pine, Douglas fir, and Oak), sample thickness (20–30 mm), and subsurface moisture depth (up to 20 mm). A prototype was then fabricated, and experimental repeated measurements confirmed high repeatability, with a standard deviation $\:\approx\:$ 0.7 dB. Calibration analysis yields an average sensitivity of 0.109 dB/%MC. These results indicate that the proposed system is suitable for wood-protective moisture-sensing applications.

## Introduction

Wood represents an affordable, renewable construction material, recognized for its high strength-to-weight ratio and much better carbon efficiency than that of other building materials^1,2^. As a natural biomaterial, wood is degradable; therefore, it is susceptible to biological degradation, which occurs much more frequently with increasing contact with water or whenever facing difficult conditions on either ground or in marine environments.

This degradation is primarily brought about by decay fungi and wood-destroying arthropods, including powderpost beetles, carpenter ants, and termites^3,4^. Most of these arthropods thrive in moist conditions. Wood with moisture content below about 24% is generally not conducive to their survival; however, long-term attack is strongly associated with moisture content of 30% and above^5^. In general, arthropods infest buildings and develop nests in water-containing wood^6^. Unlike fungal decay in situ, moisture is one of the major indications for arthropods infection, because these insects do not attack the dry wood but transport water to the nest area to sustain their activities^6,7^. Therefore, effective arthropods control strategies stress early detection of high-moisture areas in wood to allow time for repairs or replacements to be made^7^. Most methods for determining the moisture content of wood are based on one of the following techniques: weighing the wood, electrical properties testing where either resistance or capacitance is measured at various moistures, radiation-based techniques where energy loss is measured as it is transmitted through the wood, and microwave methods where changes in the dielectric constant ($\:\epsilon\:$) of infected wood are measured^8^. Among the methods of detecting moisture in wood, microwaves hold a significant position due to several advantages of the method being non-contact, safe, fast, and compatible with fixed wooden structures, and the process being non-destructive^9^. Conventional methods involve electromagnetic wave propagation characteristics, taking their transmission, reflection, and scattering for material property assessment^10^. During the last years, microwave sensors have taken significant interest, and for their realization, different kinds of antennas are under study for various sensing applications^11^. Passive antennas, like RFID tags, are based on the principle of backscattering: the incoming waves are reflected along with the modulated data^12^. On the contrary, active antennas, whose resonance frequency, reflection coefficient ($\:{S}_{11}$), or transmission coefficient ($\:{S}_{21}$) changes with variations in environmental conditions, show their response upon transmission and reception of electromagnetic waves^11–13^.

Microstrip patch sensors (MPSs) have gained widespread popularity thanks to their compactness, low cost, ease of integration with any structure, and simple fabrication process. Their versatility and compatibility with wireless technologies make them suitable for a wide range of sensing applications^14^. An annular microstrip patch resonator to measure moisture in lubricating oil is used in^15^, it can be applied for compact, non-intrusive, and accurate real-time quality monitoring applications in industrial systems. In^16,17^, various patch antennas were used to enable the non-invasive monitoring of setting times of cement paste in real-time by measuring resonant frequency changes due to variations in moisture content. Many microstrip patch sensors have also been designed for measuring the moisture content in food and agriculture applications that present efficient and reliable measurements of the moisture level in grains and mineral materials in quality and process optimization^18–23^. Near-field interference between two microstrip radiators is employed to create an absorption mechanism that allows for effective sensing of foliage moisture by detecting resonance shifts caused by the disturbance of near-field radiation from sample leaves^24^. However, most of these sensors, including resonators^25,26^, Vivaldi antennas^27^, and linearly polarized microstrip sensors^28,29^, experience considerable Tx-Rx coupling and standing-wave sensitivity that restricts reliable operation to specific antenna-to-sample distances, therefore requiring additional isolation measures or shielding. These limitations are addressed by using opposite circular polarization (CP) directions between Tx and Rx: the dry-wood specular reflection arrives cross-polarized at Rx and is inherently rejected, while moisture-induced diffuse scattering restores a co-polarized component regardless of standoff distance. To the best of the authors’ knowledge, this CP-based isolation mechanism has not been exploited in prior microwave moisture sensors for wood. Several recent works have demonstrated CP antenna designs at ISM bands using techniques such as slot perturbation^30,31^, via-assisted loading^32^, superstrate-based miniaturization^33^, and sequential feeding networks^34,35^, confirming the growing maturity of CP antenna technology. However, none of these works have explored CP antennas for material sensing applications, particularly wood moisture detection, which motivates the present study.

The current commercial devices for measuring wood moisture content have significant drawbacks. Pin-type devices require drilling into the wood, which can cause damage, while pin-less devices typically have a limited penetration depth of just 1.9 cm^36^. However, hardwoods used in homes, like certain flooring types, can be thicker than 2.54 cm^37^. Arthropods typically establish their nests below the visible wood surface^38^. An ideal pin-less solution able to detect moisture above about 30% deeper into the material would present an excellent early arthropod prevention device. In addition, practical deployment requires reliable performance across wood species, temperature variation, and subsurface moisture conditions. In general, frequency bands below 8 GHz have some advantages in sensors related to the moisture sensing application, mainly since lower frequency bands are not cross-interfered by the changes in temperature, due to the variation of dielectric properties of materials with respect to temperature. This ensures higher sensitivity and reliability even under varying ambient conditions^23^. Therefore, a long-range circularly polarized highly isolated microstrip antenna sensor for moisture detection is proposed in this paper. The proposed sensor could perform effectively at a relatively low frequency of 2 GHz. It could, in turn, distinguish effectively moist wood vulnerable to arthropods by detecting wood moisture of 30% and even higher levels. Furthermore, the proposed sensor is also able to detect moisture inside deep layers of wood and with various types of wood. CST Microwave Studio Suite performed the optimized sensor dimensions and the simulations. Finally, the proposed sensor was fabricated and the simulated results were verified by measurements using VNA.

The rest of the paper is organized as follows. Section II presents the sensing principle and system configuration. Section III presents the simulated sensor characteristics. Section IV presents the experimental results and discussion. Section V outlines limitations and future work, and Section VI concludes the paper.

**Fig. 1:**
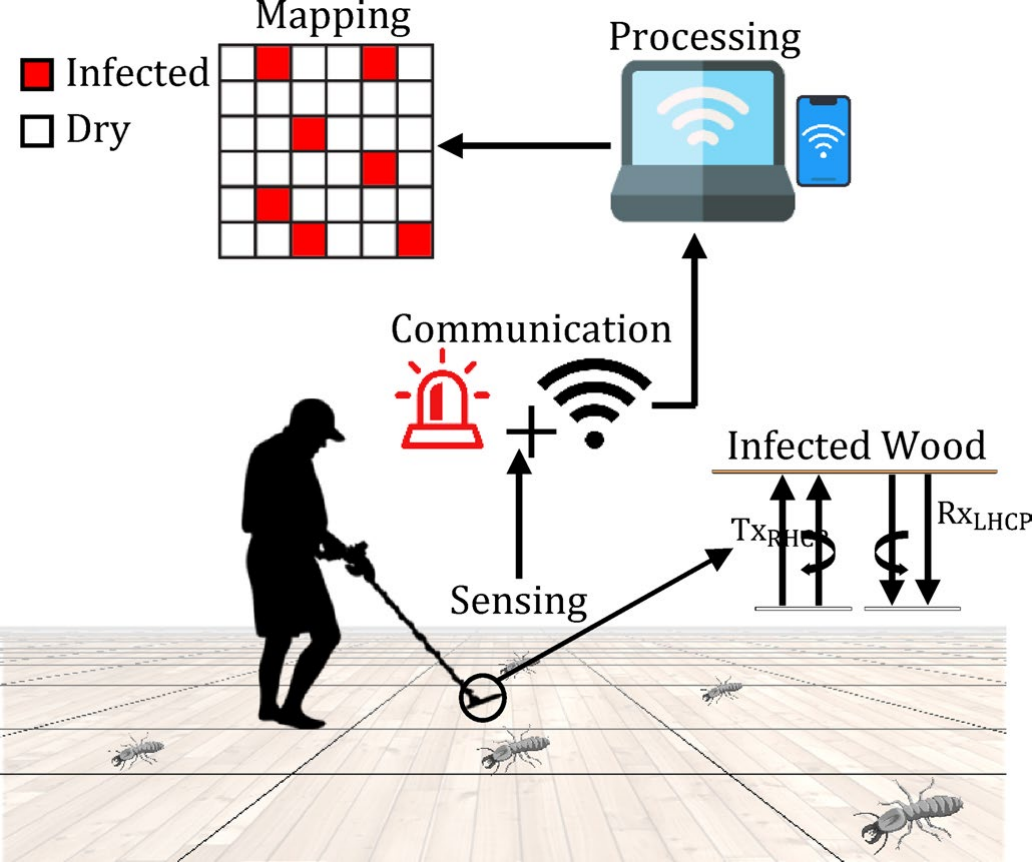
Arthropod infestation detection using a circularly polarized antenna sensing system.

### Proposed sensing system

A. *Sensing Principle*.

The main concept of the proposed sensing system is the difference in wood relative permittivity resulting from the corresponding different moisture level that in turn influences the reflection of electromagnetic waves^23^. With the increasing level of humidity, the relative permittivity increases and thus there is an increased amount of electromagnetic reflections from the wood surface^39^. Moreover, increased moisture content not only increases the electrical conductivity of wood significantly but also leads to more reflections^40–42^. This observation is intimately associated with the high relationship between the interface reflection coefficient of two media and the impedance of the medium, which is characterized as a function of its conductivity and dielectric constant, as expressed by Eqs. (1) and (2)^43^.

1 $$\:{\Gamma\:}=\frac{{Z}_{2}-{Z}_{1}}{{Z}_{2}+{Z}_{1}}$$

2 $$\:Z=\sqrt{\frac{j\omega\:\mu\:}{\sigma\:+j\omega\:\epsilon\:}}$$

Where $\:{Z}_{1}$ is the impedance of air, $\:{Z}_{2}$ is the impedance of the wood, $\:\epsilon\:\:$is the permittivity, $\:\sigma\:$ is the conductivity, $\:\mu\:\:$is the permeability, and $\:\omega\:=2\pi\:f$ is the angular frequency. Equations (1) and (2) are used as a first-order interface model assuming a locally planar air to wood boundary, near normal incidence, and homogeneous effective material parameters. Although wood is anisotropic and its surface is not perfectly smooth, we employ scalar dielectric properties at 2 GHz based on the material characterization in Table 2, and any residual orientation dependent and roughness dependent deviations are treated as practical uncertainty as reflected by the repeatability results reported in Section IV. Wood permittivity is known to vary with fiber orientation; however, at 2 GHz the transverse grain direction, which is the dominant orientation for flat structural panels, yields well-characterized scalar values as tabulated in Table 2, and this scalar approximation has been validated experimentally in the 0.75–2.5 GHz band^44^. As moisture content increases, both the effective permittivity and conductivity increase, which lowers the wood impedance $\:{Z}_{2}$ in Eq. (2) and increases the reflected coefficient $\:{\Gamma\:}$ in Eq. (1). The moist wood surface therefore scatters more power toward the Rx antenna. Since the Tx and Rx antennas use opposite CP direction, only scattered power that carries a co-polarized component (produced by diffuse scattering from the lossy moist wood) is effectively received, while the specular dry-wood reflection is rejected. The net result is an increase in |S₂₁| with moisture content, reaching a maximum difference of 12.5 dB between dry (MC = 0%) and fully moist (MC = 100%) wood at 2 GHz, as shown in Section III-A. In the ideal case (infected wood as a perfect conductor with infinite conductivity), the impedance is close to zero, leading to the full reflection of electromagnetic waves, for linearly polarized waves the reflected wave direction is reversed. As a result, the situation generates a standing wave with its maximum amplitude at positions computed by Eq. (3)^45^.

3 $$\:{d}_{max}=\frac{(n+1)\lambda\:}{4}$$

Where $\:n$ is an integer, and $\:\lambda\:$ is the wavelength. This results in a limitation at which distances the system works efficiently^29^, which is mitigated using the proposed CP system, since specular reflection reverses the CP direction, suppressing the reflected field at the Rx port regardless of standoff distance. Operating frequency has been found to be a significant parameter of the observed wood dielectric constant value, increasing with decreasing operating frequency values^45^. In order to determine the appropriate operating frequency, the microwave penetration depth $\:\delta\:$ into the wood material was derived in Eq. (4)^45^.

4 $$\:\delta\:=\frac{{\lambda\:}_{0}}{\pi\:\sqrt{\epsilon\:{\prime\:}}\mathrm{tan}\delta\:}$$

Where $\:\mathrm{tan}\delta\:=\frac{\epsilon\:{\prime\:}{\prime\:}}{\epsilon\:{\prime\:}}$, is the dielectric loss tangent (dimensionless), is valid under the low-loss condition $\:\left({\epsilon\:}^{{\prime\:}{\prime\:}}\ll\:{\epsilon\:}^{{\prime\:}}\right)$ which is generally satisfied for wood at microwave frequencies. At higher frequencies, the penetration depth decreases, limiting sensing to surface effects, while at very low frequencies the antenna becomes impractically large.

**Fig. 2:**
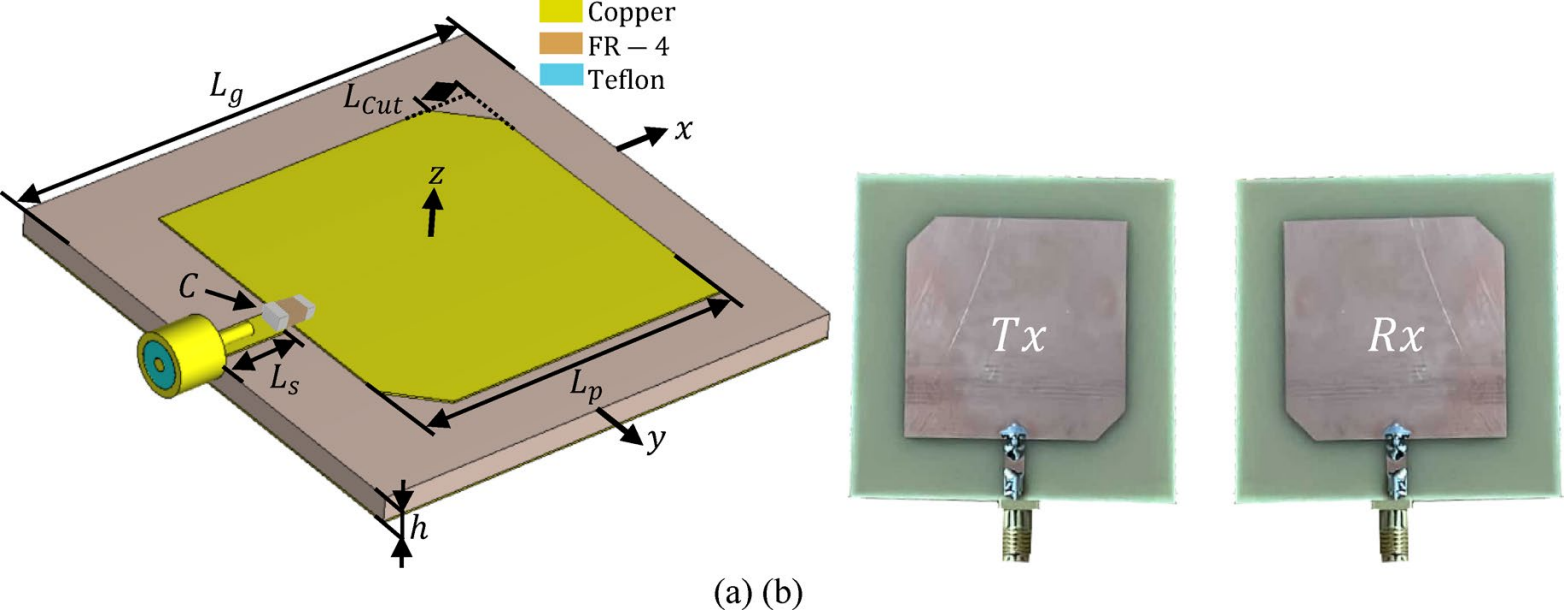
Circularly polarized microstrip antenna used in the proposed sensing system. (a) Simulated antenna layout. (b) Fabricated Tx and Rx prototypes.

For wood, typical dielectric parameters at microwave frequencies are in the range $\:{\epsilon\:}^{{\prime\:}}\approx\:2\:to\:3.5$ and, $\:{\epsilon\:}^{{\prime\:}{\prime\:}}\approx\:0.2\:to\:0.4$, yielding penetration depths on the order of several centimeters at 2 GHz^46^. Recent experimental studies have measured real and imaginary parts of complex permittivity for wood in the 0.75–2.5 GHz band, confirming both the suitability of this frequency range for bulk moisture sensing and the expected variation in dielectric behavior^44^. Therefore, 2 GHz was selected as a well-justified compromise, providing sufficient penetration depth while maintaining practical antenna dimensions. The free-space wavelength at 2 GHz ($\:\lambda\:$ = 150 mm) does not allow sub-centimeter nest localization; rather, the system functions as an area-averaged moisture alarm over the antenna footprint (~50 mm), and spatial mapping can be achieved by scanning the sensor over multiple positions on the wood surface, as described in Section IV.

B. *System Configuration*.

**Fig. 3:**
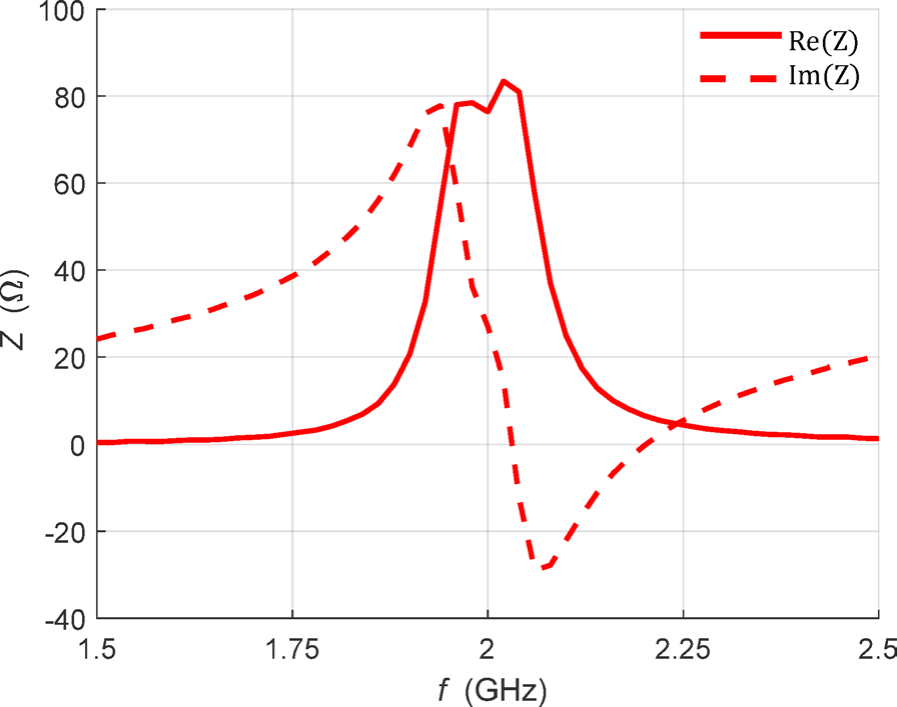
Characteristic impedance $\:Z$ of the circularly polarized microstrip patch.

**Fig. 4:**
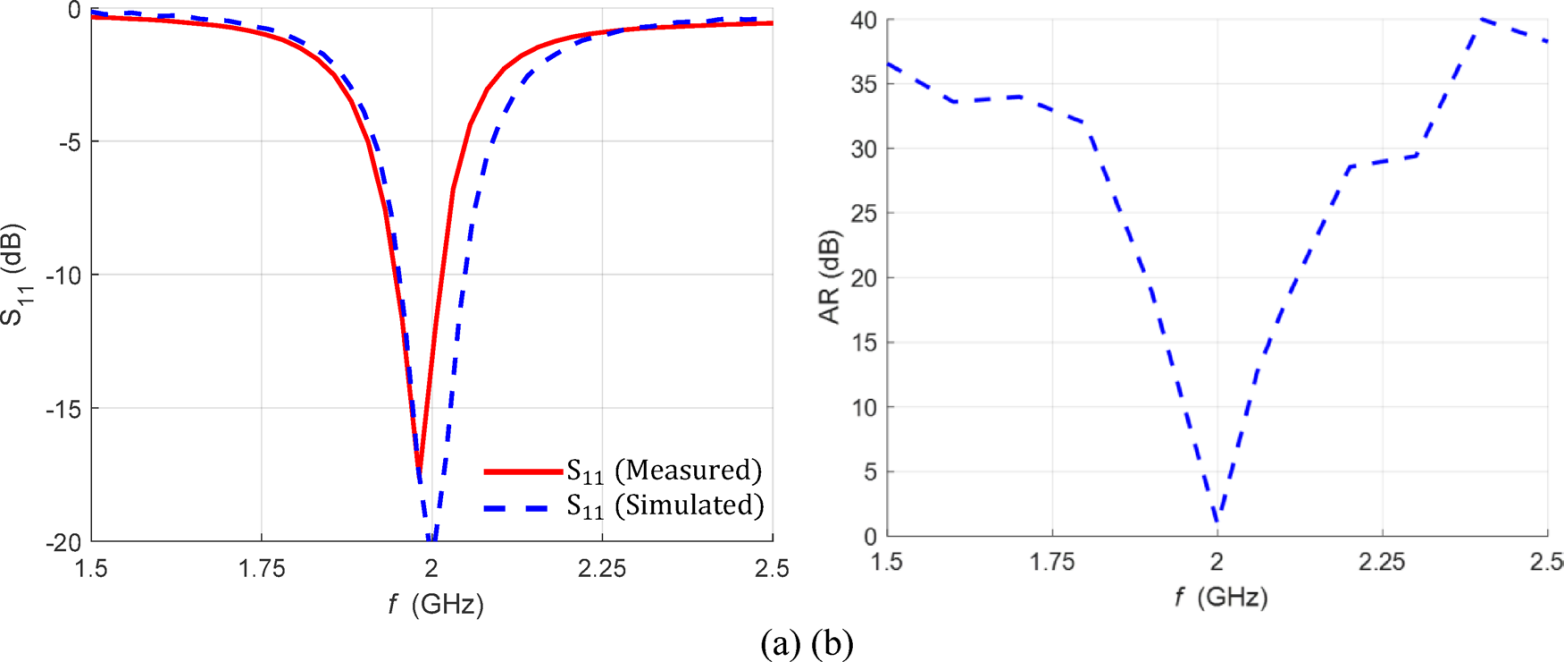
Proposed antenna results over frequency (a) Reflection coefficient$\:\:{S}_{11}$, (b) Axial ratio (AR).

The intended field-use scenario of the proposed circularly polarized antenna sensing system for non-invasive inspection of wooden structures is illustrated in Fig. 1. The sensing system employs two CP microstrip antennas, one operating as a transmitter (Tx) and the other as a receiver (Rx) that collects the wood-reflected response. Using opposite circular polarization directions between Tx and Rx provides high isolation in the absence of strong reflections in the case of dry wood, while moisture-affected wood increases the received response. The antenna layout and fabricated prototype are shown in Fig. 2. The antennas are fabricated on a grounded FR4 substrate with relative permittivity $\:{\epsilon\:}_{r}=4.4$, loss tangent $\:\mathrm{tan}\delta\:=0.019$, and thickness $\:h=1.6\:mm$. The main antenna design parameters are listed in Table 1. These parameters were optimized to achieve circular polarization at 2 GHz, which is the selected operating frequency of the proposed sensing system. The stub, ground plane, and microstrip patch are made from copper-coated layers 0.035 mm thick. FR4 is widely adopted in many areas and therefore easily accessible, cheap, and strong enough^47^. In addition, it has high resistance against moisture and heat stress, especially in the closed space of high humidity and high temperature^48^. Since both Tx and Rx antennas are fabricated on the same substrate batch and operated under identical ambient conditions, any FR4 dielectric drift with temperature or humidity acts as a common-mode effect that does not alter the differential |S₂₁| sensing metric. At the beginning, the antenna presented inductive impedance characteristics as shown in Fig. 3. To improve matching to 50 Ω feed line, a 3-pF capacitor was employed. The simulated and measured reflection coefficient $\:{S}_{11}$ is shown in Fig. 4(a) and indicates narrowband operation around 2 GHz. Moreover, the antenna exhibits an axial ratio (AR) of 0.93 dB at 2 GHz, as shown in Fig. 4(b), confirming that the corner truncation (.

= 4.7 mm) successfully excites two orthogonal modes ($\:T{M}_{10}$ and $\:T{M}_{01}$) with equal amplitude and 90° phase difference which is the condition required for CP. The opposite CP direction between Tx and Rx causes the dry-wood specular reflection to arrive at Rx cross-polarized and be rejected (~ − 39 dB), while moisture-induced scattering restores a co-polarized component, producing the observed 12.5 dB increase in |$\:{S}_{21}$|.

## Sensor characteristics

In this section, the performance of the proposed system was studied under different conditions such as using different wood types, temperature, moisture content, and effect of the receiving antenna orientation. The fundamental sensing principle of the proposed system is based on moisture-driven changes in the reflected and scattered microwave field from the wood sample. Therefore, the sensing metric adopted in this work is the magnitude of the transmission coefficient $\:\left|{S}_{21}\right|$ at the fixed operating frequency of 2 GHz, which represents the overall Tx to Rx system response, rather than the individual antenna reflection coefficient$\:\:\left|{S}_{11}\right|$. Initially, in this experimental setup, the separation distance between the antenna and the wood sample had to be varied to obtain highest reflection. Distance between the Tx and Rx antennas was optimized for the infected wood case to 150 mm in order-.

**Table 1:** Antenna design parameters.

Parameters	Value
$\:{\boldsymbol{L}}_{\boldsymbol{g}}$	$\:50\:mm$
$\:{\boldsymbol{L}}_{\boldsymbol{p}}$	$\:35\:mm$
$\:{\boldsymbol{L}}_{\boldsymbol{C}\boldsymbol{u}\boldsymbol{t}}$	$\:4.7\:mm$
$\:{\boldsymbol{L}}_{\boldsymbol{s}}$	$\:8\:mm$
$\:{\boldsymbol{W}}_{\boldsymbol{s}}$	$\:3.11\:mm$
$\:\boldsymbol{h}$	$\:1.6\:mm$
$\:{\boldsymbol{\epsilon\:}}_{\boldsymbol{r}}$	$\:4.4$
$\:\boldsymbol{C}$	$\:3\:pF$

-to reduce mutual coupling between the two elements and maximize the received power. A wood sample with dimensions of 200 mm × 150 mm × 25 mm was used to test the proposed sensing system. The electrical properties of wood used in our simulation setup at 2 GHz are shown in Table 2 for dry and infected wood, assuming the electric field is perpendicular to the wood fibers^47^.

C. *Moisture Content*.

**Fig. 5:**
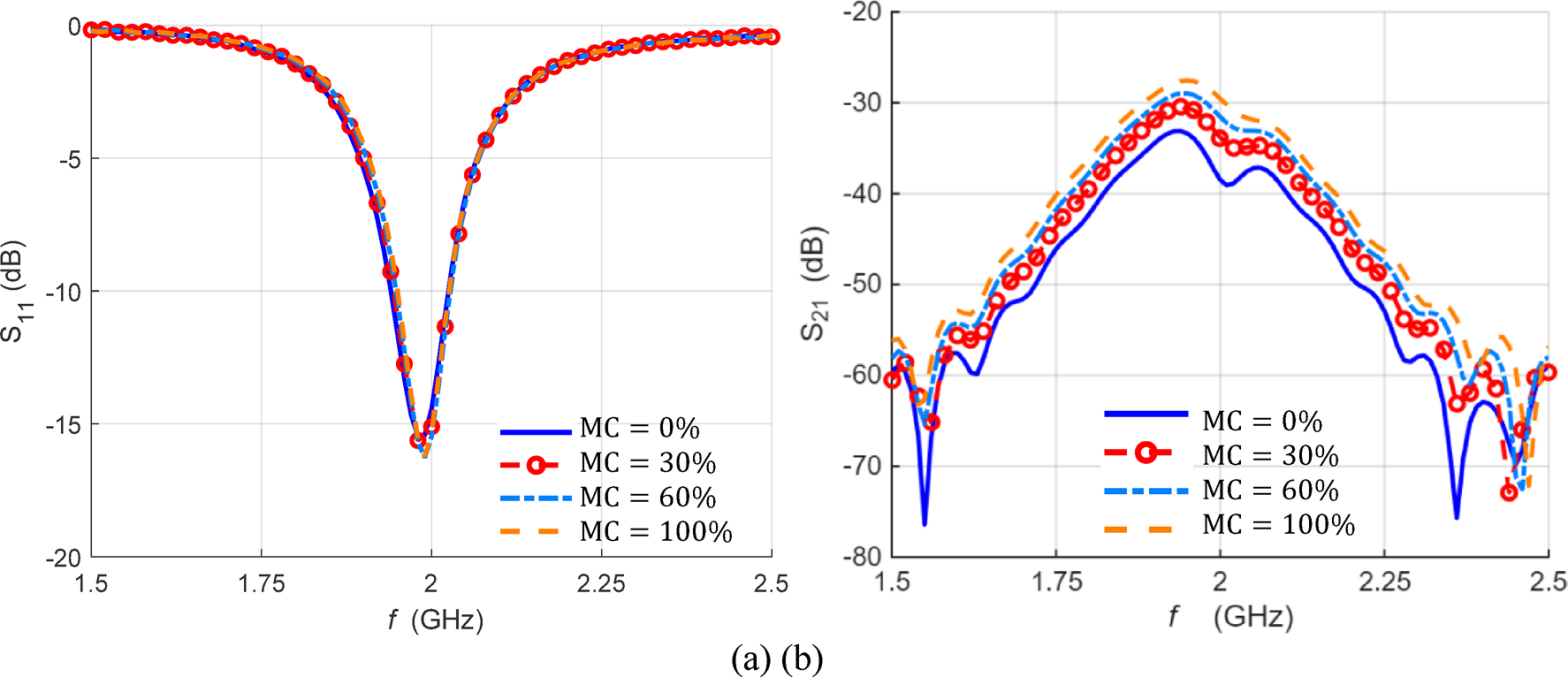
Comparative plots of simulated system characteristics for various moisture contents of Oak wood. (a) Reflection coefficient$\:\:{S}_{11}$, (b) Transmission coefficient$\:\:{S}_{21}$.

To extensively study the proposed moisture sensor reliability, various levels of wood moisture were investigated in Fig. 5. As the moisture content (MC) increases from the dry state to fully infected (i.e., MC = 100%) where Oak wood characteristics change according to Table 2, the electrical conductivity of the wood increases substantially, and more reflected power from the wood surface is transmitted to the Rx side, resulting in an increase in the value of $\:{S}_{21}$. Compared to dry case, the proposed sensor efficiently detects changes in wood moisture with differences exceeding 12.5 dB at the resonance frequency of 2 GHz. Differentiating between various moisture states (i.e., MC = 30, 60, and 100%), with differences exceeding 3.4 dB among the different wood moisture levels.

**Table 2:** Electrical properties of different wood types with different MC.

Wood Type	Pine		Douglas fir		Oak	
Case	Parameters	Value	Parameters	Value	Parameters	Value
Dry Wood MC (0%)	$\:{\epsilon\:}_{r}\:$	$\:1.63$	$\:{\epsilon\:}_{r}\:$	$\:1.73$	$\:{\epsilon\:}_{r}\:$	$\:2.23$
	$\:{tan}\delta\:\:$	$\:0.02\:$	$\:{tan}\delta\:\:$	$\:0.02$	$\:{tan}\delta\:\:$	$\:0.03\:$
Infected Wood MC (30%)	$\:{\epsilon\:}_{r}\:$	$\:3.4$	$\:{\epsilon\:}_{r}\:$	$\:4.06$	$\:{\epsilon\:}_{r}\:$	$\:5.8$
	$\:{tan}\delta\:\:$	$\:0.18\:$	$\:{tan}\delta\:\:$	$\:0.22\:$	$\:{tan}\delta\:\:$	$\:0.35$
Infected Wood MC (60%)	$\:{\epsilon\:}_{r}\:$	$\:5.39$	$\:{\epsilon\:}_{r}\:$	$\:6.61$	$\:{\epsilon\:}_{r}\:$	$\:10.3$
	$\:{tan}\delta\:\:$	$\:0.15\:$	$\:{tan}\delta\:\:$	$\:0.2\:$	$\:{tan}\delta\:\:$	$\:0.31$
Infected Wood MC (100%)	$\:{\epsilon\:}_{r}\:$	$\:10.29$	$\:{\epsilon\:}_{r}\:$	$\:12.89$	$\:{\epsilon\:}_{r}\:$	$\:18.17$
	$\:{tan}\delta\:\:$	$\:0.14$	$\:{tan}\delta\:\:$	$\:0.14$	$\:{tan}\delta\:\:$	$\:0.3$

**Fig. 6:**
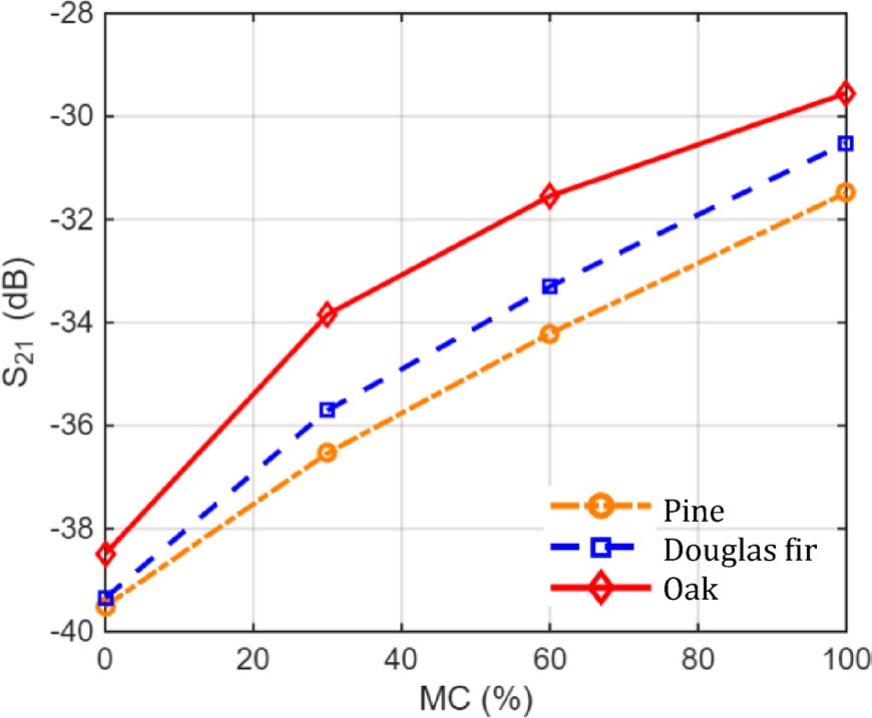
Simulated transmission coefficient$\:\:{S}_{21}$at 2 GHz versus moisture content (MC) for Pine, Douglas fir, and Oak.

D. *Wood Type*.

To study the effectiveness of the proposed sensor for detecting moisture across different wood types, various hardwood and softwood samples such as Oak, Douglas fir, and Pine were investigated, as shown in Fig. 6. The density values for Pine, Douglas fir, and Oak are 0.4, 0.5, and 0.8 g/cm³, respectively^47^, the density values affect the wood characteristics as shown in Table 2. Figure 6 illustrates the simulated transmission coefficient $\:{S}_{21}$ at 2 GHz versus moisture content for Pine, Douglas fir, and Oak. The results show that, for the same MC level, higher-density wood (Oak) produces a larger $\:{S}_{21}$ increase compared with softwoods, consistent with the larger moisture-driven changes in dielectric properties listed in Table 2. The proposed sensor can efficiently detect the moisture content in different types of softwood and hardwood. Additionally, the dependence of dielectric properties on wood type and density, as well as the operating frequency, affects the reflection, absorption, and transmission of electromagnetic waves^29^.

E. *Temperature*.

**Fig. 7:**
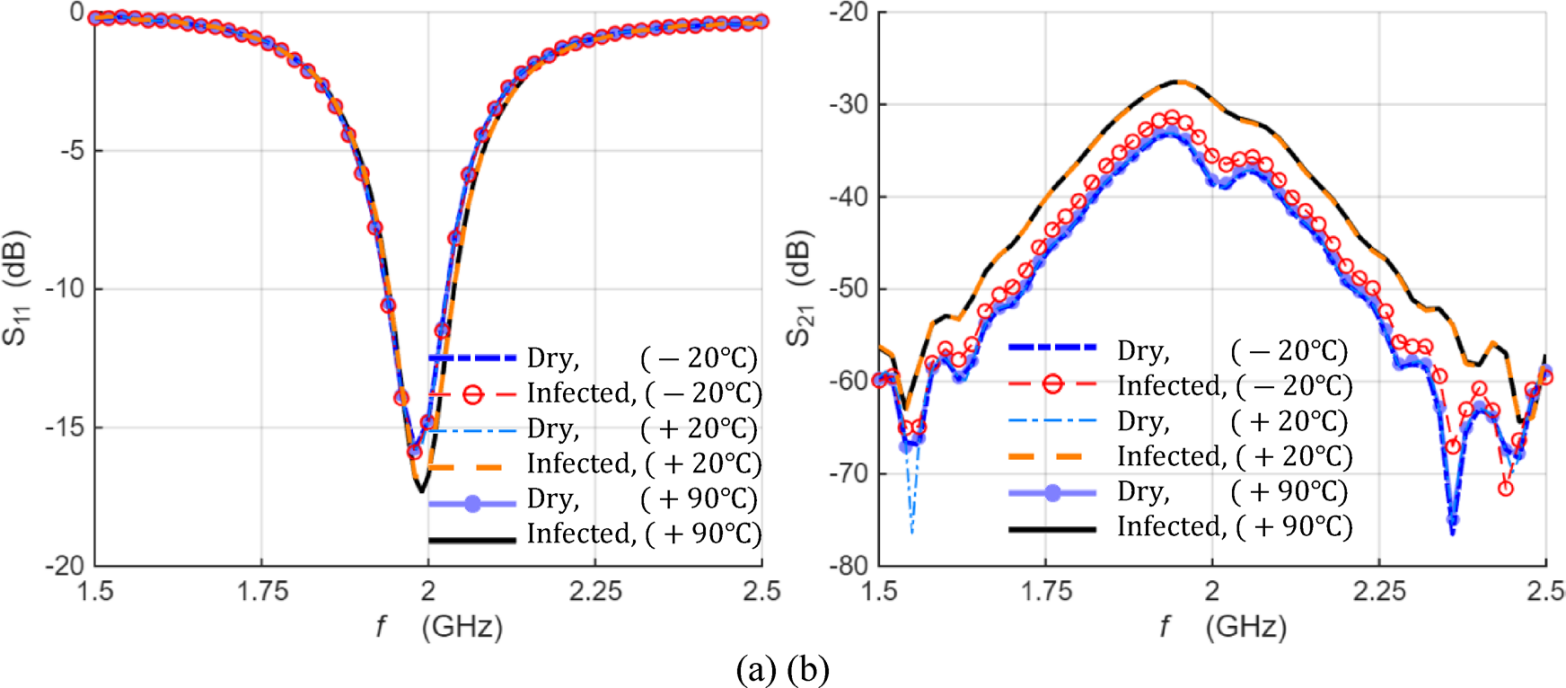
Comparative plots of simulated system characteristics for various temperature degrees of Oak wood. (a) Reflection coefficient$\:\:{S}_{11}$, (b) Transmission coefficient$\:\:{S}_{21}$.

To further investigate the reliability of the proposed moisture sensor, various temperature degrees were studied in Fig. 7. As the temperature varies from $\:-{20}^{o}C\:$to $\:{+90}^{o}C$ where wood characteristics change according to Table 3 for Oak wood type^49^. Figure 7(a) shows that the antenna matching $\:{S}_{11}$ remains nearly unchanged across the considered temperatures, for both dry and fully infected cases, confirming stable antenna operation around 2 GHz. Figure 7(b) shows that the transmission response $\:{S}_{21}$ exhibits only limited temperature dependence compared with the strong moisture-driven change, and the separation between dry and fully infected wood remains clearly distinguishable at 2 GHz for all three temperatures (− 20 °C, 20 °C, and 90 °C). At sub-zero temperature, water in wood can partially freeze, which reduces effective dielectric properties, therefore the infected-case dielectric parameters differ from room-temperature values^47^.

**Table 3:** Electrical properties of oak wood at different temperature degrees in different cases.

Case	Parameters	Value
Dry Wood MC (0%), T (-$\:2{0}^{\boldsymbol{o}}$C)	$\:{\epsilon\:}_{r}\:$	$\:2.13$
	$\:{tan}\delta\:\:$	$\:0.02\:$
Infected Wood MC (100%), T (-$\:2{0}^{\boldsymbol{o}}$C)	$\:{\epsilon\:}_{r}\:$	$\:4.11$
	$\:{tan}\delta\:\:$	$\:0.32$
Dry Wood MC (0%), T (+$\:2{0}^{\boldsymbol{o}}$C)	$\:{\epsilon\:}_{r}\:$	$\:2.23$
	$\:{tan}\delta\:\:$	$\:0.03\:$
Infected Wood MC (100%), T (+$\:2{0}^{\boldsymbol{o}}$C)	$\:{\epsilon\:}_{r}\:$	$\:18.17$
	$\:{tan}\delta\:\:$	$\:0.3$
Dry Wood MC (0%), T (+$\:9{0}^{\boldsymbol{o}}$C)	$\:{\epsilon\:}_{r}\:$	$\:2.43$
	$\:{tan}\delta\:\:$	$\:0.04\:$
Infected Wood MC (100%), T (+$\:9{0}^{\boldsymbol{o}}$C)	$\:{\epsilon\:}_{r}\:$	$\:18.17$
	$\:{tan}\delta\:\:$	$\:0.23$

Hence, the proposed sensor is reliable through different temperature degrees and efficiently detects changes in wood moisture with differences exceeding 12.5 dB at the resonance frequency of 2 GHz. Differentiating between various moisture states (i.e., MC = 0, and 100%).

F. *Receiver Orientation*.

Due to the antenna’s directional characteristics, its sensitivity to the received signal depends on its relative alignment to the Tx antenna, and the measured sample.

**Fig. 8:**
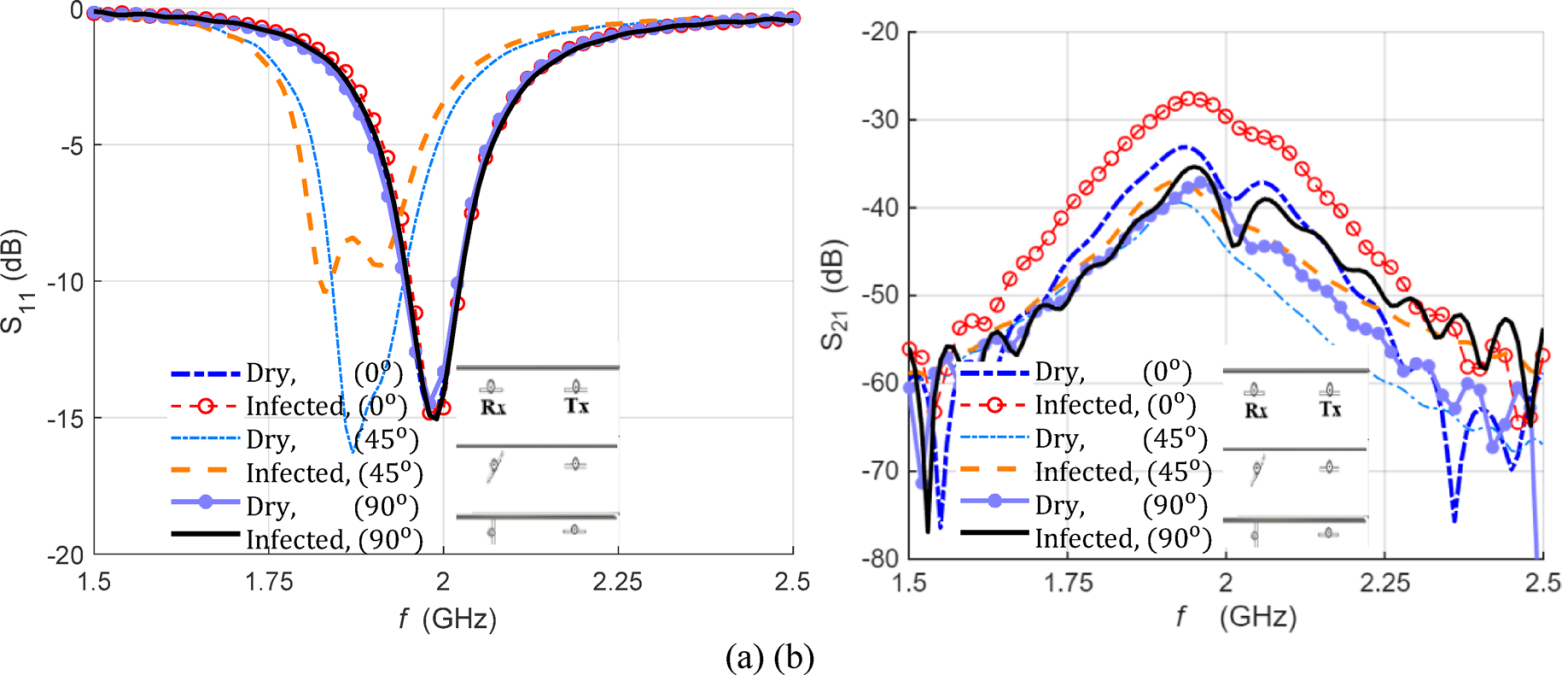
Comparative plots of simulated system characteristics at different orientation for Rx antenna. (a) Reflection coefficient$\:\:{S}_{11}$, (b) Transmission coefficient$\:\:{S}_{21}$.

**Fig. 9:**
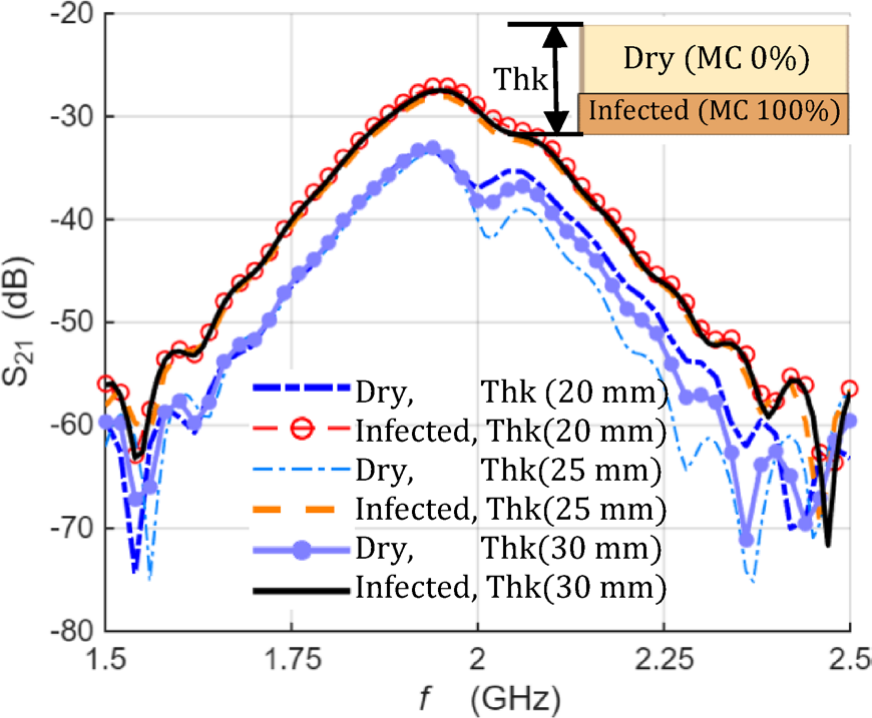
Simulated transmission coefficient$\:\:{S}_{21}$at for Oak wood in the dry (MC 0%) and infected (MC 100%) cases at different sample thickness values.

**Fig. 10:**
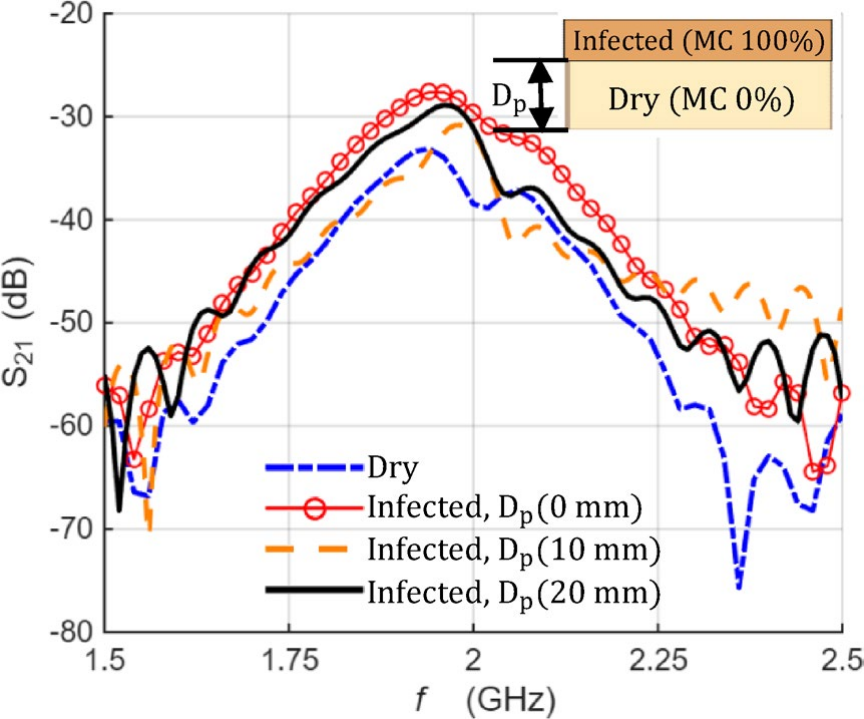
Simulated transmission coefficient$\:\:{S}_{21}$at for Oak wood in the dry (MC 0%) reference case and for a moisture-affected layer (MC 100%) located at different depths$\:\:{\mathrm{D}}_{\mathrm{p}}$ beneath a dry surface layer.

This affects the reflection coefficient slightly at certain angles such as $\:{45}^{o}$as illustrated in Fig. 8(a). However, as the Rx antenna is rotated, its position relative to the Tx antenna and the wood sample changes. This alteration affects the angle at which the Rx antenna receives the signal from the Tx antenna and the reflected signal from the wood, which in turn influences the amount of transmitted power to the Rx side, as shown in Fig. 8(b). However, for practical purposes, the best adjustment is achieved when the Rx antenna is aligned horizontally.

G. *Wood Thickness*.

To study the effect of the wood sample thickness on the proposed sensing system, the Oak sample thickness was varied in the simulation setup while keeping the antenna geometry, separation distance, and dielectric parameters fixed for both dry (MC 0%) and infected (MC 100%) cases. Figure 9 shows that varying the thickness from 20 mm to 30 mm produces only a minor change of 1.2 dB in the magnitude of $\:{S}_{21}$ around the operating frequency of 2 GHz, while the separation between dry and infected cases remains clearly distinguishable. This behavior indicates that the proposed system response is mainly governed by the near-field interaction with the wood surface region, and therefore increasing the thickness beyond this effective interaction depth results in a limited additional change in$\:{\:S}_{21}$.

H. *Measurement Depth*.

Since arthropods tend to live beneath the surface of wood and the wood surface is more likely to be dry compared to the inner layers, in this section, we investigated the capability of the proposed sensor to penetrate a dry surface and detect moisture in the underlying wood layers. Figure 10 shows that as the moisture depth $\:{\mathrm{D}}_{\mathrm{p}}$ increases from 0 mm to 20 mm, the difference in $\:{\:S}_{21}$ between the partially moist wood and the fully dry reference decreases, which is expected due to the near-field nature of the proposed system where the field intensity and the associated scattered response decay with depth inside lossy media. However, the moisture-induced contrast remains clearly observable even at $\:{\mathrm{D}}_{\mathrm{p}}=20\:mm$, confirming that the proposed sensor can still detect moisture located below a dry surface layer with a sufficient margin. At 2 GHz, the extracted $\:{\:S}_{21}$ contrast relative to the dry case remains 6.78 dB for $\:{\mathrm{D}}_{\mathrm{p}}=20\:mm$ (compared to 8.94 dB at $\:{\mathrm{D}}_{\mathrm{p}}=0\:mm$), which is significantly larger than the observed standard deviation level reported in Section IV, and therefore enables reliable discrimination between dry wood and subsurface moisture.

## Results and discussions

**Fig. 11:**
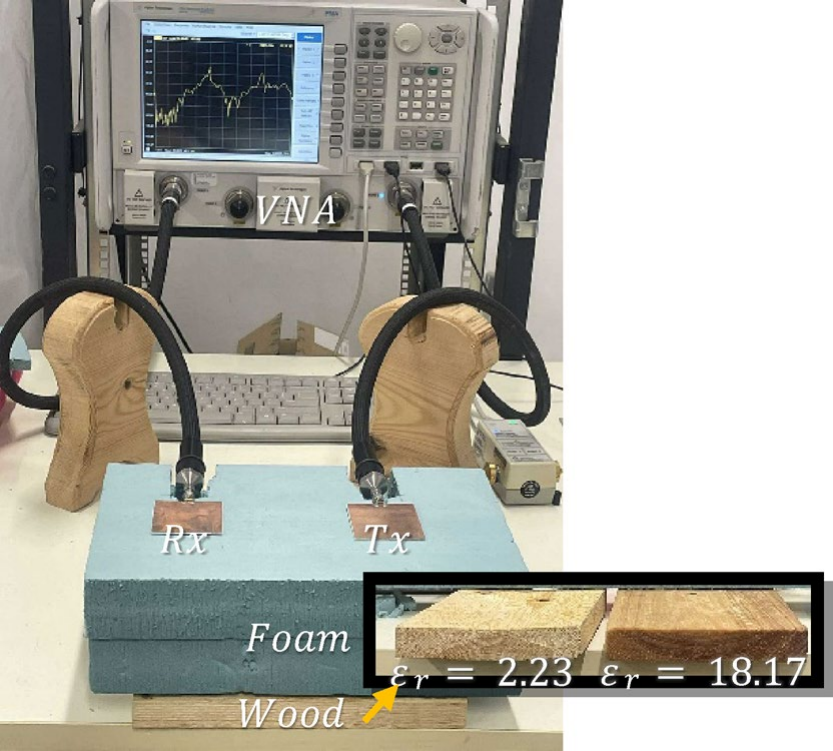
Measurement setup for the proposed sensing system.

In this section, the performance of the proposed moisture sensing system was studied by investigating variations in both reflection and transmission coefficients. Using two scenarios, one with both antennas having the same polarization direction providing complete isolation and the other with antennas having opposite polarization directions providing high power transfer, a high agreement between simulated and measurement results was obtained. The experimental environment setup is shown in Fig. 11. A single Oak wood sample with dimensions of 200 mm × 150 mm × 25 mm was used to evaluate the proposed sensor indoors at room temperature.

**Fig. 12:**
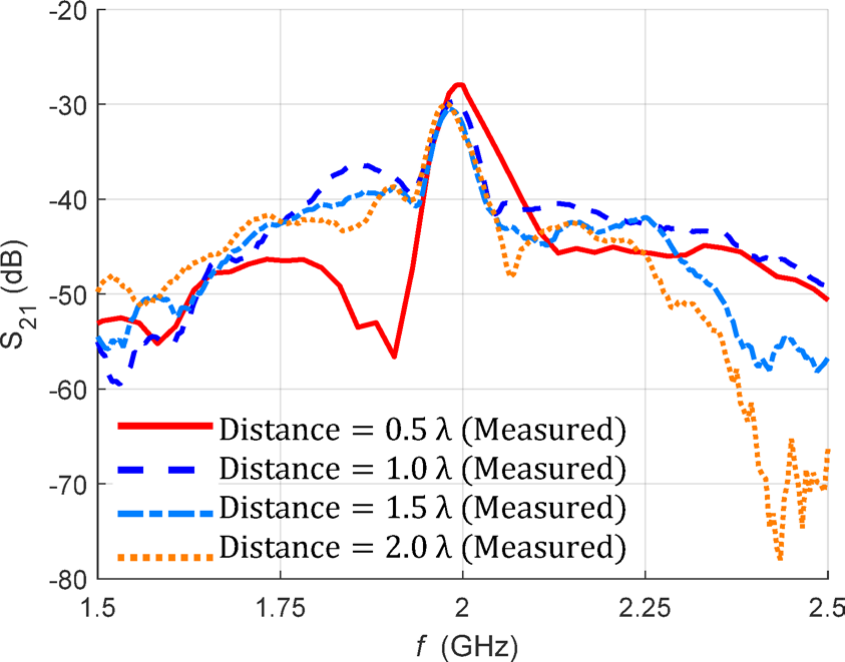
Comparative analysis of the measured transmission coefficients$\:\:{S}_{21}$ at different distances between the antenna system and the moisture-affected wood sample.

**Fig. 13:**
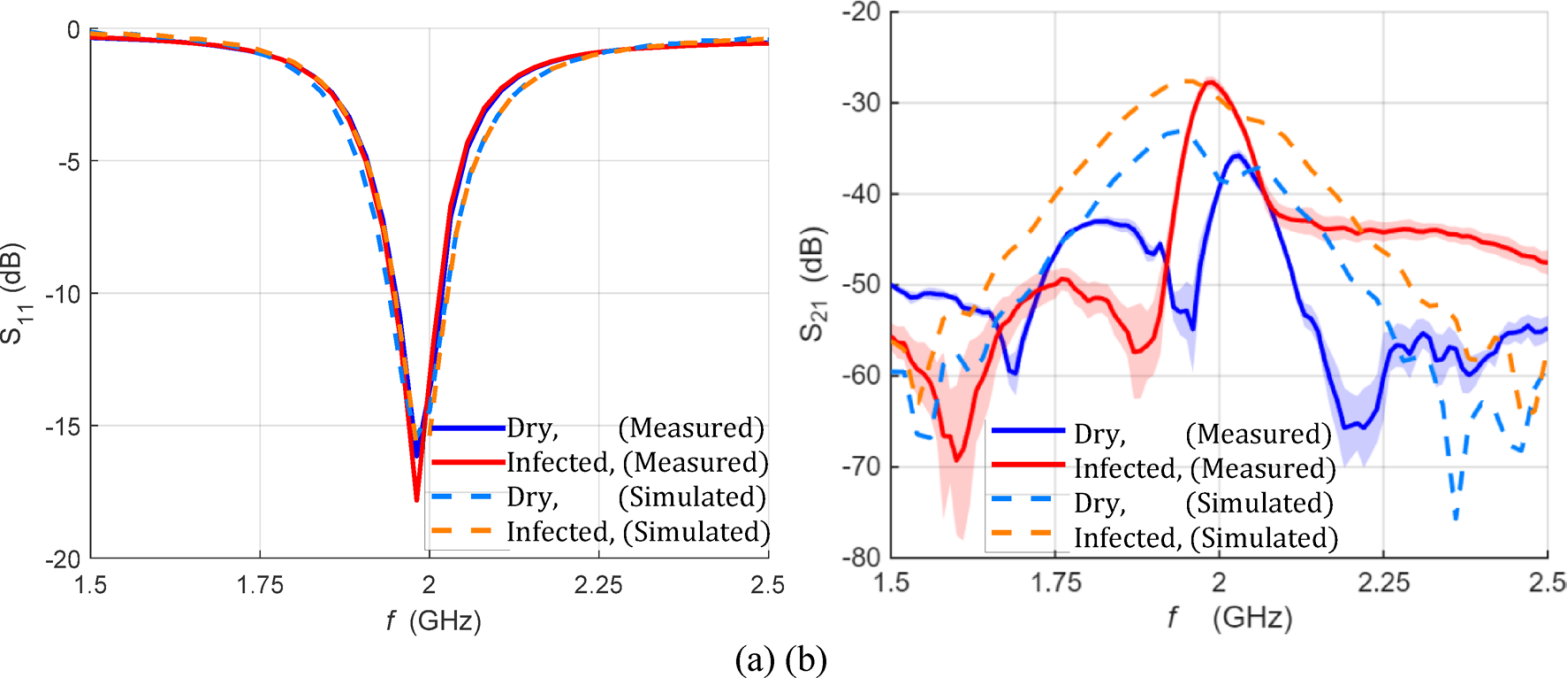
Proposed system simulated and measured results in the case of circular polarization with different directions (a) Reflection coefficient$\:\:{S}_{11}$, (b) Transmission coefficient$\:\:{S}_{21}$.

The wood sample was tested as one piece placed in front of the Tx/Rx antenna pair, and the reported responses represent the effective interaction over the illuminated area of the sample. For the reported ‘dry’ and ‘infected reference measurements, the wood sample was conditioned to achieve an approximately uniform moisture state prior to measurement (dry: oven-dried; infected: water-conditioned) and sealed to reduce moisture loss during the test. A distance of 0.5$\:\lambda\:$ was selected for further analysis. Layers of foam were used to separate the wood from the sensor with an optimized distance (d) of approximately 75 mm. In practical cases, moisture may be spatially non-uniform; in that case, localized infected regions can be detected by scanning the sensor over multiple positions to construct a moisture map. The effect of the distance between antenna and wood sample on system performance was also investigated. Only a minor effect of about 2 dB when the distance is increased by a factor of four, as shown in Fig. 12. This is due to the behavior of the wood sample as a secondary radiator that re-radiates the incident wave toward the receiving antenna. For a sample dimension of 200 mm, the far-field distance at 2 GHz is approximately 533 mm, while the measurements were performed at distances of 0.5$\:\lambda\:$–2$\:\lambda\:$ (75–300 mm). This shows that the receiving antenna operates within the near-field of the wood radiator, where the simple $\:1/{R}^{2}$ path-loss dependence is not applicable. However, the received field results from near-field scattering and aperture coupling. In addition, this near-field behavior demonstrates the robustness of the proposed sensing system, since reliable performance can be maintained without strict control of antenna-to-sample spacing. For the moisture-sensing test, a comparison between both dry and infected wood samples was investigated and validated. The primary objective of the proposed system is the detection of arthropod infestation, rather than precise humidity calibration. As shown in Fig. 13, the maximum difference between the “infected” and “dry” cases is approximately 12.5 dB, which is sufficiently large to enable reliable detection of infestation. Finally, for reflection and transmission coefficient measurements, the Keysight N5227A vector network analyzer was used. To ensure the repeatability and robustness of the proposed sensing system, both the infected and dry cases were measured ten times under the same conditions. For each case, the responses over the frequency band were measured, and the standard deviation was calculated at each frequency point.

I. *First Case*.

In this case, both the transmitter and receiver are circularly polarized with opposite polarization directions. Figure 13 shows Measured and simulated results for the reflection and transmission coefficients. Solid curves represent the mean of ten repeated measurements, with shaded regions indicating ± 0.7 dB, ± 0.76 dB at 2 GHz standard deviation for dry and infected wood, respectively. Dashed curves correspond to full-wave simulations.

J. *Second Case*.

**Fig. 14:**
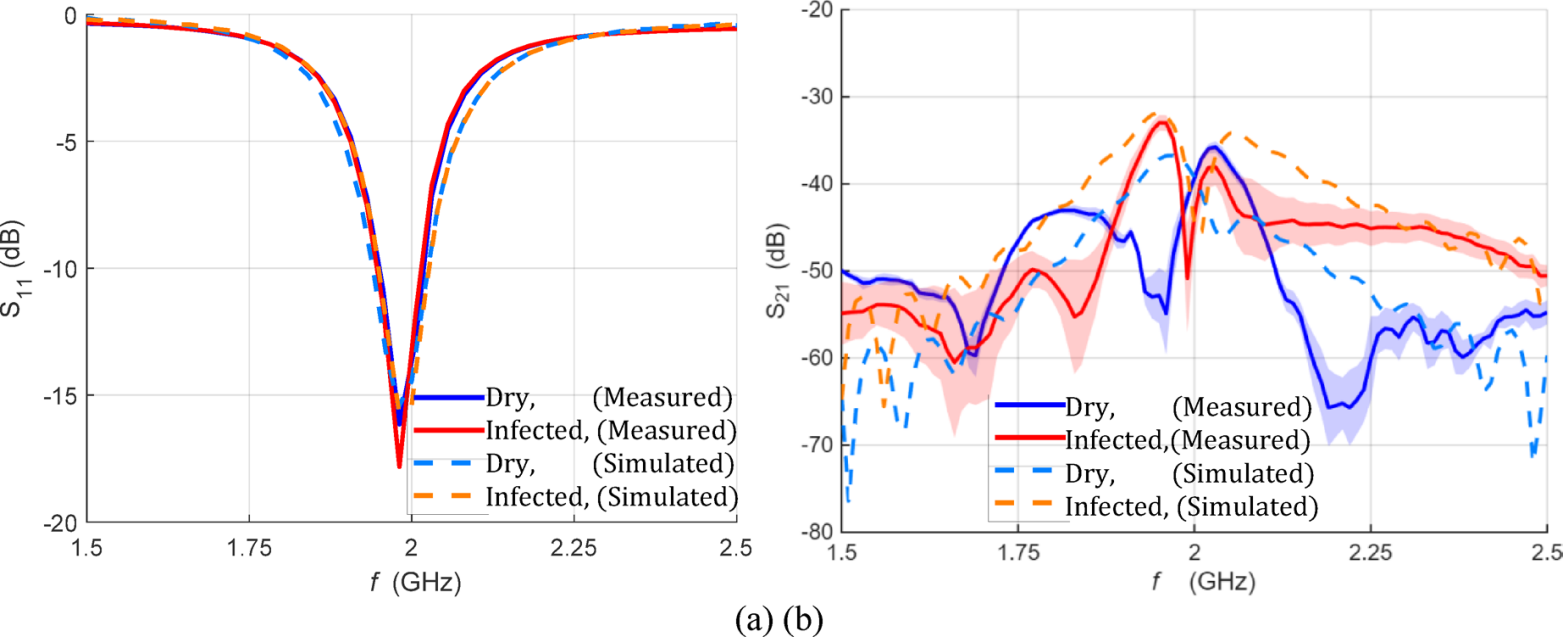
Proposed system simulated and measured results in the case of circular polarization with the same directions (a) Reflection coefficient$\:\:{S}_{11}$, (b) Transmission coefficient$\:\:{S}_{21}$.

In this case, both the transmitter and receiver are circularly polarized but with the same polarization directions. Figure 14 shows Measured and simulated results for the reflection and transmission coefficients. Solid curves represent the mean of ten repeated measurements, with shaded regions indicating ± 3.3 dB, ± 0.76 dB at 2 GHz standard deviation for dry and infected wood, respectively. Dashed curves correspond to full-wave simulations. Still, the reflection coefficient is not affected in all cases. However, the transmission coefficient in this case provides a dip value at 2 GHz in the case of infected wood.

**Fig. 15:**
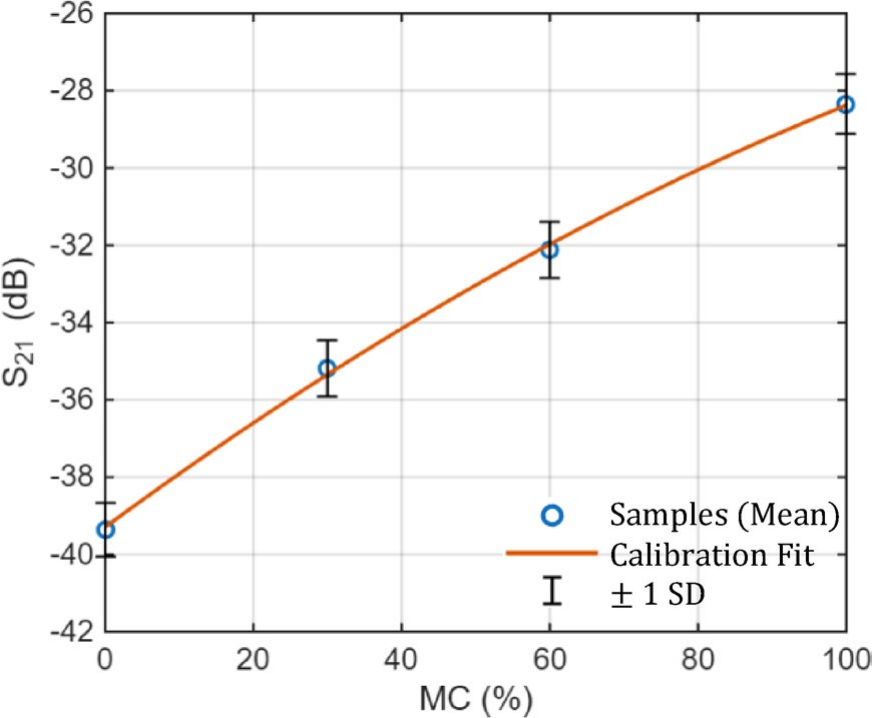
Calibration curve of $\:\left|{S}_{21}\right|$ at 2 GHz versus moisture content (MC).

**Table 4:** Measurement of oak wood with different MC.

MC (%)	$\:{\:\mathbf{M}\mathbf{e}\mathbf{a}\mathbf{n}\:(\mathbf{S}}_{21}\:\left(\mathbf{d}\mathbf{B}\right))$	$\:\mathbf{S}\mathbf{D}\:\left(\mathbf{d}\mathbf{B}\right)$
0	−39.35	0.7
30	−35.16	0.72
60	−32.12	0.73
100	−28.34	0.76

Which verifies the effectiveness of the circular polarization at this frequency and can be used as a second step of verification on the infected wood structures, the simulation results also agree with this result with a slight difference caused by the same issues mentioned before.

K. *Calibration Curve and Sensitivity*.

To quantify the sensor performance, calibration measurements at 2 GHz were extracted from the opposite-polarization case (highest sensitivity), as this configuration provides optimal signal contrast between dry and moist wood. Figure 15 shows the calibration curve of $\:\left|{S}_{21}\right|$ at 2 GHz versus moisture content (MC), and the measured calibration points (mean ± SD, *N* = 10) are listed in Table 4. To enable quantitative use of the proposed system as a sensor, the extracted calibration points were fitted using a second-order polynomial:

6 $$\:{y}_{dB}=-0.00032{x}^{2}+0.1412x-39.292$$

where $\:x$ is moisture content (MC%) and $\:{y}_{dB}\:$is$\:\:\left|{S}_{21}\right|$ in dB. the fit quality is quantified by an RMSE of 0.123 dB with respect to the mean measured points reported in Table 4 and plotted in Fig. 15. Sensitivity is defined as the slope of the calibration curve (dB/%MC); using Table 4, the average sensitivity is 0.109 dB/%MC for MC = 0–100%. Using the reported standard deviation (SD) as the $\:\left|{S}_{21}\right|$ variability at each MC level, the moisture tolerance (± 1σ) is approximately ± 5.9 MC at MC = 30% and ± 7.1 MC at MC = 60%.

Table 5 provides a comparison between the proposed system and other microwave moisture proposed systems in the literature. This comparison shows that the proposed antenna provides a wide range of moisture content measuring with a max difference of 12.5 dB between the minimum and maximum values. In good agreement, the antenna operation represented by the reflection coefficient is almost unaffected in all cases. However, the transmission coefficient shows a difference of around 12.5 dB between the two samples in both simulated and measured results, which is desired to easily differentiate between the two cases. A slight deviation could result from inaccuracies during the measurement setup or from manufacturing errors. Additionally, surrounding objects in the real environment can affect the electromagnetic signals.

## Limitations and future work

The proposed system shows good performance in arthropod infestation detection in wood. However, some limitations remain to be noted. First, scalability of the approach to industrial level monitoring is yet to be addressed. Second, although the sensing performance was evaluated across different wood types and over a wide temperature range, long-term stability against varying environmental conditions, i.e., ambient humidity, aging, and outdoor deployment effects still remains to be tested. Third, while simulations indicate limited sensitivity to thickness variation and detectable subsurface moisture up to 20 mm, deeper or more complex moisture distributions may reduce contrast and should be further investigated. Future work will try to address such limitations through research on optimized antenna design with reduced power and enhanced sensitivity, compact hardware integration, and scalable array configurations for large-scale deployment. In addition, new signal processing and machine learning methods will be investigated for achieving maximum environmental change resistance and auto-classification of infestation intensity. Finally, miniaturization design as well as integration of IoT-based connectivity will be pursued for maximizing the practical, long-term field application of the sensor system proposed.

**Table 5:** Comparison between different moisture sensors.

Ref.	Structure	$\:{\boldsymbol{f}}_{\boldsymbol{r}\boldsymbol{e}\boldsymbol{s}}$ (GHz)	Max Difference (dB)	MC Range (%)	Environment	Sensitivity (dB/%MC)	Size ($\:{\boldsymbol{\lambda\:}}^{2}$)
^25^(2021)	Resonator	6	15	NA	Contact	NA	0.56 × 0.56
^26^(2022)	Resonator	3–6	NA	0 : 100	Contact	NA	0.21 × 0.18
^27^(2023)	Vivaldi	6–12	NA	26 : 67	Free space	NA	2 × 0.8
^28^(2024)	Microstrip	5.8	10	46 : 54	Free space	1.25	0.68 × 0.68
^29^(2025)	H-Slot	3.35–4.58	8.3	0 : 100	Free space	0.23	0.28 × 0.28
^50^(2025)	Circular Microstrip	2.45	17	2 : 11	Contact	1.4	0.49 × 0.49
^51^(2026)	Waveguide + probe antenna	2.45	NA	52 : 75	Near field	NA	1.5 × 0.75
This work	**CP Microstrip**	**2**	**12.5**	**0 : 100**	**Free space**	**0.109**	**0.33 × 0.33**

## Conclusion

In this article, we presented the design and performance evaluation of a wood moisture sensor targeting arthropods control. The proposed sensor is a dual-antenna sensing system based on the microwave technique. For high isolation performance, the antennas were designed to be circularly polarized and it enabled verification of moisture detection using two reception scenarios (same and opposite circular polarization direction). This also allowed long distance measuring. The sensor was fabricated on cheap commercial FR4, with dimensions of 50 mm × 50 mm × 1.6 mm. The antenna system operates effectively at 2 GHz, providing highly circular polarized radiation with an axial ratio of 0.93 dB and mutual coupling below − 38 dB in the uninfected scenario. On the other hand, in the infected scenario, the S-parameters of the proposed sensor were investigated in different polarization direction cases. While $\:{S}_{11}$ remained nearly unchanged, the transmission response $\:{S}_{21}$ increased by up to 12.5 dB at 2 GHz in moisture-affected wood, enabling reliable discrimination from dry wood. Performance was verified across different wood species and practical operating variations (environmental and setup-related), showing robust moisture discrimination. The system covered 0–100% moisture content with limited temperature dependence over 20–90 °C, and the horizontal Tx–Rx alignment provided the most consistent sensitivity. Higher-density wood exhibited stronger $\:{S}_{21}$ contrast, but moisture discrimination remained clear across the tested species. The sensor demonstrates reliable moisture detection at depths up to 20 mm beneath dry surface layers, a crucial capability for early arthropod detection, with measured $\:{S}_{21}$ contrast remaining 6.78 dB at 20 mm depth, which is significantly larger than measurement standard deviation and enables reliable discrimination between dry and subsurface moisture.

## Data Availability

The data that support the findings of this study are available from the corresponding author upon reasonable request.
